# Feasibility and Impact of Trauma-Informed Care Training in Internal Medicine Residency: A Pilot Study

**DOI:** 10.7759/cureus.22368

**Published:** 2022-02-18

**Authors:** Deepa Ramadurai, Julie Knoeckel, Roger J Stace, Sarah Stella

**Affiliations:** 1 Pulmonary and Critical Care Medicine, Hospital of the University of Pennsylvania, Philadelphia, USA; 2 Leonard Davis Institute of Health Economics, University of Pennsylvania, Philadelphia, USA; 3 Hospital Medicine, Denver Health and Hospitals, Denver, USA; 4 Business School, University of Colorado Denver, Denver, USA; 5 Internal Medicine, University of Colorado Anschutz Medical Campus, Denver, USA

**Keywords:** interactive workshop, internal medicine residency, curriculum development and evaluation, trauma informed care, medical resident education

## Abstract

Introduction: Mounting evidence indicates that early life trauma is highly prevalent and associated with adverse health outcomes later in life. However, primary care providers report lacking the training to effectively address trauma encountered in daily practice. There is a paucity of research describing the implementation and evaluation of trauma-informed care (TIC) curricula within Graduate Medical Education.

Methods: We piloted a three-hour TIC workshop facilitated by a community-based psychologist expert to assess the feasibility and impact of TIC training on Internal Medicine (IM) residents’ knowledge, attitudes and skills related to TIC. Participants were a subset of IM residents in a health-equity-focused curricular pathway in the University of Colorado IM Residency. Residents completed anonymous surveys one week before and after the workshop, and a final survey 10 weeks later. Residents who did not participate in the workshop completed a similar baseline survey (control group). Data were analyzed using matched pair T-tests.

Results: Fourteen of 20 residents (70%) who participated in the pilot workshop completed the initial survey. Of these, 10 (71%) completed the first post-workshop survey, and seven (50%) completed the final survey. We observed significant improvements in residents’ self-reported knowledge, attitudes and skills related to TIC. The majority of residents in the control group reported a desire for TIC training.

Conclusions: TIC is an important curricular gap in IM training. A single, brief TIC workshop was feasible and was associated with improved self-reported knowledge, attitudes and skills among IM residents.

## Introduction

According to the Substance Abuse and Mental Health Services Administration, individual trauma “results from an event, series of events, or set of circumstances [which are] physically or emotionally harmful or life-threatening with lasting adverse effects on… functioning and mental, physical, social, emotional, or spiritual well-being” [1]. Trauma is associated with poor health outcomes, including decreased access to care, and increased morbidity and mortality across a broad range of health conditions [2-5]. In the landmark adverse childhood experiences (ACEs) study, more than 50% of adults reported experiencing at least one ACE while 6% reported four or more. ACEs include any psychological and physical abuse or exposure to household dysfunction including substance abuse, mental illness, violent treatment of a family member, and criminal behavior [4].

Trauma is increasingly recognized as a widespread public health problem, with early life adversity identified as a driver of high-cost medical complexity [6]. Trauma-informed care (TIC) is a framework for recognizing, understanding, and acknowledging patients' adverse life experiences by emphasizing compassion and avoidance of re-traumatization. A Journal of the American Medical Association perspective called for a multifaceted approach to mitigate the impact of trauma on individuals and on the health care system. This includes a “universal precautions” approach to screening and TIC training for health care providers, establishing TIC as a best practice in comprehensive care [7].

There is a high incidence of early life trauma among adult primary care patient populations [8-10]. Low-income, underserved communities are particularly susceptible. A history of trauma is almost universal among patients with mental health and substance use disorders, those experiencing homelessness, military veterans, those with a history of incarceration, and among refugees [5,11-16]. Additionally, patients may be traumatized through health care interactions, including hospitalization or routine medical procedures [17]. Despite this, primary care providers (PCPs) feel inadequately trained to address trauma [18].

While some medical schools have introduced TIC curricula, implementation of TIC training within Graduate Medical Education (GME) has been limited [19-24]. Residents in the University of Colorado Internal Medicine Residency Program (IMRP) frequently care for patient populations likely to have high prevalence of trauma, however, there was no dedicated TIC curriculum prior to 2018. We partnered with a community expert to develop an innovative TIC workshop to address this gap in a subset of IMRP residents.

## Materials and methods

Setting and participants

The University of Colorado IMRP consists of approximately 170 residents who rotate at four clinical sites: a university hospital, a public safety-net hospital, Veterans Affairs (VA), and a community hospital. Residents select one of three curricular pathways based on academic interests in their second year: the Health Equity Pathway (HEP), Research Pathway, and the Medical Education Pathway. The HEP, created in 2016, aims to improve residents’ understanding of social determinants of health (SDOH) and equip them with the skills to provide clinical care and advocate for patient populations who are more likely to experience health disparities. Residents in the HEP (approximately one-third of second- and third-year residents) have a portion of their academic half-days dedicated to health equity-focused lectures and workshops (see Appendix A for content details). The HEP partners with community members in curriculum development and content delivery.

The TIC workshop was held during a required academic half-day for the HEP during the 2018-2019 academic year. Learning objectives (Table 1) were distributed to the residents prior to the workshop via email. The pilot study was reviewed and approved by the Colorado Multiple Institutional Review Board.

**Table 1:** Learning Objectives for Trauma Informed Care Workshop

Learning Objectives
1. Define trauma and differentiate between different types: developmental, single event trauma, cultural and intergenerational.
2. Describe the impact of trauma on patients seeking medical care.
3. Understand and apply information from the Adverse Childhood Experience (ACE) Study in interviewing and examining a patient with a history of trauma.
4. Learn practical tools to aid in fostering connection, empowerment, hope and sensitivity for patients who have experienced trauma.
5. Understand the impacts of vicarious trauma and compassion fatigue on healthcare providers, and tips for addressing this.

Description of intervention

The pilot intervention consisted of a single, three-hour workshop with a community-based psychologist expert who provided TIC training for staff at the Colorado Coalition for the Homeless [25]. The HEP workshop was adapted from this training and included a didactic PowerPoint presentation, which defined trauma and its impacts on brain, body and behavior, followed by a "how to" session, which simulated patient encounters and cases in an open-format small-group discussion. Residents practiced terminology to use with patients who may have experienced trauma, and our psychologist expert provided feedback on how certain phrases may be perceived by a patient who has experienced trauma from her own experience. Residents recalled their own patient interactions related to trauma and delivered peer-to-peer feedback.

Data collection and analysis

Twenty residents attended the TIC workshop and were invited to participate in an anonymous, voluntary pre-workshop survey emailed via Qualtrics. There was no incentive for participation nor penalty if they did not participate. The pre-survey was emailed one week prior to the workshop, with reminder emails the day prior and morning of the workshop. There were two post-workshop surveys: one emailed to the group one week following the workshop, and a second emailed to the group 10 weeks following the workshop (see Appendix B for HEP pre- and post-surveys). Only those who completed the pre-workshop survey were asked to complete the post-workshop and 10-week surveys. All surveys were developed and approved by the authors following a literature review, with questions categorized by as primarily representing knowledge, attitudes, or skills of TIC.

To protect anonymity, each subject was assigned a unique identification number associated with their email address. Data were de-identified prior to analysis. Only the investigators had access to the results. Analysis of pre- and post-workshop surveys with matched pair T-tests was performed independently for each domain: knowledge, attitudes, or skills.

A separate cohort of 83 second- and third-year residents who were not in the HEP and did not participate in the TIC workshop received a survey to assess knowledge, attitudes and skills with respect to TIC and their desire for TIC training (non-HEP survey, Appendix C).

The last two questions of the HEP surveys and the last question of the non-HEP survey utilize free-text responses. These were reviewed by the authors to gain further insight into resident perception of the workshop.

## Results

The number of surveys completed and description of participants are shown in Table 2. Fourteen of 20 HEP residents (70%) completed the pre-workshop survey. Of those who completed the initial survey, 11 (71%) completed the post-workshop survey, and seven (50%) completed the final survey. Additionally, 23 of 83 (28%) second- and third-year non-HEP residents completed surveys.

**Table 2:** Characteristics of workshop participants from the Health Equity Pathway and those not in the Health Equity Pathway

Pathway	Survey Completed	Total (%)	PGY-2 (%)	PGY-3 (%)	Gender, male (%)
HEP	Pre-Workshop	14 (70)	5 (35.7)	9 (64.3)	9 (64.3)
HEP	Post-Workshop	11 (71)	5 (50)	5 (50)	7 (70)
HEP	Final (after 10 weeks)	7 (50)	2 (28.6)	5 (71.4)	5 (71.4)
Non-HEP	N/A	23 (27.7)	14 (60.9)	9 (39.1)	13 (56.5)

HEP residents who completed the survey were majority male (64.3%) and third-year residents (64.3%). None had received previous TIC training at the time of the baseline survey. Four (28.6%) were aware of the ACE study, and none had experience administering it in a clinical setting.

Prior to the workshop, residents reported that trauma was highly prevalent in the patients they cared for (mean 4.5), and that trauma impacts an individual’s ability to seek medical care (mean 4.6) (Table 3). Residents agreed patients are not personally responsible for the trauma they experience (specifically patients with substance use disorders, mean 4.2), and this was strengthened following the workshop (mean 4.7).

**Table 3:** Comparison of Health Equity Pathway (HEP) residents’ pre-, post- and final surveys. Mean values are shown here, however data analysis (paired samples T-test) was performed at the individual level. Mean responses from non-Health Equity Pathway (non-HEP) residents are also demonstrated here for comparison. (Likert response scale: 1 = strongly disagree, 2 = disagree, 3 = neither agree nor disagree, 4 = agree, 5 = strongly agree; unless otherwise noted*)

Survey Question (grouped by knowledge, attitudes, and skills designation)	HEP Pre-Workshop Response (mean)	HEP Post-Workshop Response (mean)	HEP Final Response (mean)	Non-HEP Response (mean)
Knowledge				
Trauma is prevalent among patients I treat.	4.5	4.8	5.0	4.2
I can identify different types of trauma (e.g., developmental, single event trauma, cultural and intergenerational).	2.4	3.8	3.4	2.6
Trauma is distinct from everyday stress.	4.4	4.6	4.9	4.4
Trauma impacts an individual’s ability to seek medical care.	4.6	4.7	4.9	4.6
I know what resources are available for patients after discussing his/her history of trauma, including coping strategies.	2.2	3.1	3.1	2.2
Adverse life circumstances are likely to be responsible for a patient’s trauma.	3.6	4.1	4.1	4.0
Attitudes				
Patients who experience trauma have challenging medical and social issues which I can learn from.	4.1	4.4	4.4	4.4
Patients are personally responsible for the trauma they experience (e.g., substance use).*	4.2	4.7	4.3	3.8
Patients who experience trauma frequently over-utilize health care resources.	3.4	3.7	3.4	3.6
Caring for patients with trauma is difficult and leads to burnout and compassion fatigue.	3.6	3.7	3.6	3.6
Patients who have experienced trauma may have difficulties adhering to medical therapies as prescribed.	4.1	4.5	4.6	4.2
Patients who leave medical care against medical advice might be exhibiting the effects of trauma.	4.0	4.6	4.7	4.3
Skills				
I can identify specific instances where an understanding of trauma-informed care is useful in patient care.	3.3	4.3	4.3	4.0
I am comfortable inquiring about physical, emotional, and sexual abuse in my patients.	2.3	3.3	3.9	3.0
I am able to recognize signs and symptoms of trauma, even if my patient does not disclose them to me.	3.0	3.8	3.9	2.8
I routinely encourage patients to disclose what traumatic experiences they feel comfortable sharing.	2.0	2.3	2.9	2.7
I routinely ask patients how they cope with emotional responses that may result from traumatic experiences.	2.4	2.4	2.7	2.8

Six non-HEP residents had previously heard of the ACE study (26%), one of whom had previously administered it. One non-HEP resident had received TIC training in the past. There were no significant differences between HEP pre-workshop versus non-HEP residents’ knowledge, attitudes, and skills.

**Figure 1:**
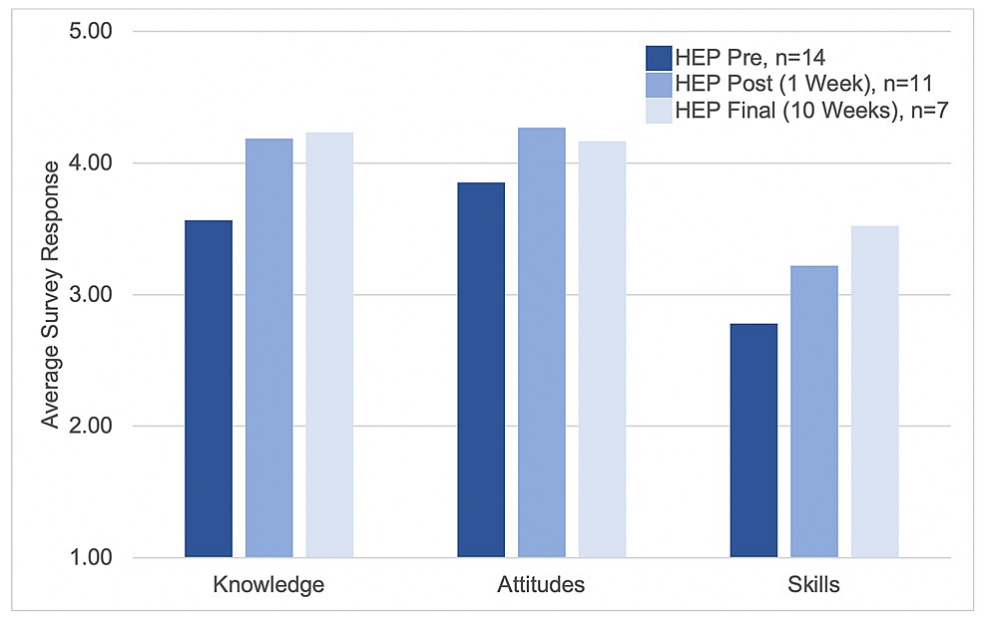
Mean Likert scale responses before and after a Trauma-Informed Care Workshop for Internal Medicine residents categorized by their knowledge, attitudes, and skills HEP: Health Equity Pathway

Between the pre-workshop and post-workshop surveys, we observed statistically significant differences in all composite domains for individual residents (Figure 1): self-reported knowledge (t=2.3, p<0.024), trauma-informed attitudes (t=2.54, p<0.016), and skills (t=2.71, p<0.012). These improvements were sustained on the final survey at 10 weeks: knowledge, t=3.46, p<0.007; attitudes, t=2.64, p<0.019; skills, t=2.18, p<0.036.

HEP residents cited a number of different “most important” takeaways from the TIC workshop and desired more practical training (Table 4).

**Table 4:** Residents’ open-ended feedback responses

Question	Response
What was the most important thing you learned from the Trauma-Informed Care workshop?	“How to recognize past trauma in patient's even when it is not something they directly report”
	“Learning about the ACE study, which I had never heard of.”
	“[Learning] new ways to ask for permission”
	“Trauma informed care could be an orientation like patient-centered care”
	“How to talk to patients in order to make them feel more comfortable in medical settings”
What further training would you like to receive on this topic?	“More practical training on addressing trauma in the primary care setting”
	“Specific strategies for obtaining trauma history and how to address issues such as chronic pain from a trauma-informed perspective”
	“Samples on how to address these issues in practice”

## Discussion

TIC is a curricular gap in IM and other primary care-oriented residency training programs [22]. While residents in our pilot study strongly agreed that trauma was prevalent in the patients they cared for, only one of the 36 residents surveyed (HEP and non-HEP) reported having any previous training in TIC, supporting the presence of this curricular gap. Moreover, fewer than 30% of the HEP residents had ever heard of the ACE study, and few understood the distinction between various types of trauma. Hence, defining trauma and a basic introduction to the ACE survey were crucial within this workshop.

Few studies have evaluated TIC curricula in GME, and only one included IM residents [23,24]. Shamaskin-Galloway and colleagues reported that a pilot TIC curriculum, consisting of five hours of didactics, a 10-minute reflection, and an optional feedback session, was associated with increased self-reported knowledge, attitudes and practices around TIC amongst trainees in an interprofessional primary care residency [24]. A TIC curriculum for pediatric residents demonstrated interactive role-playing exercises that improved residents' trust in and partnership with mothers of neonates with neonatal abstinence syndrome [23].

While TIC may be viewed as beyond the purview of IM, internists frequently provide care for medically complex patient populations suffering the adverse effects of trauma. A qualitative study by Green and colleagues suggests that PCPs lack the knowledge and specialized skills needed to meaningfully address the trauma they encounter in their daily practice [18]. Lack of formal training, in combination with the strong emotional reactions that patients who have experienced trauma may evoke, creates conflict and undermines physicians’ effectiveness. This perpetuates health disparities among patients who are victims of trauma and physicians may subsequently experience secondary traumatic stress [26].

Teaching TIC aligns with the Accreditation Council of Graduate Medical Education (ACGME) mandate to incorporate patient-centered care, ethics, professionalism, and humanism into GME [27]. Introducing TIC during residency allows trainees to practice delivering TIC and provides the opportunity to obtain feedback from patients and supervising providers. A topic discussion by Ravi et al. [28] identifies seven arenas of the outpatient visit where TIC skills may be implemented. Implementation of a case-based workshop in this setting in IMRPs could then include TIC competency as a GME requirement. Although our TIC workshop was associated with improved self-reported skills, these skills were implemented with low frequency and low confidence. Further efforts such as role-play, case-based simulation, and objective structured clinical exam are needed to bolster TIC skills.

Our study has a few limitations. First, the intervention was studied in a subset of second- and third-year residents in a large, academic IMRP who elected to participate in the HEP. Thus, our findings may not be generalizable to other non-academic IMRPs and may be influenced by selection bias, both in the HEP group who is more likely to identify a positive impact of the TIC workshop and widened by the selection bias in the control group who is less likely to identify a positive impact of a TIC workshop. We did not re-survey the control group after 10 weeks to determine if the self-perceived improvement in knowledge, attitudes and skills was in fact related to the workshop rather than a result of furthering their internal medicine training. However, since TIC is rarely discussed in the context of internal medicine [24], we believe this effect would have been small if present. In addition, sample sizes were small, as only a portion of residents are in the HEP, although, this is typical for studies of educational interventions in GME including previously attempted TIC education in residency programs [22-24]. The response rate of the intervention group was not 100% and decreased on subsequent surveys. Individuals who believe strongly in the importance of TIC may have been more likely to respond and respond favorably.

Our pilot study adds to the small body of research describing the implementation and evaluation of TIC curricula in GME. By utilizing matched pairs T-tests, we attempted to mitigate selection bias. The 10-week survey was intended to evaluate sustainability of results. Since TIC training is broadly applicable to GME and interprofessional education, IMRPs may consider partnering with other departments or community organizations to offset costs and enhance sustainability as we believe partnership with a community expert was key to the design and delivery of content. Lastly, our pilot study provided preliminary data on feasibility and generated preliminary data to guide further curriculum development. We have utilized our findings from this pilot study to develop a tailored TIC workshop which was delivered to all interns as well as HEP upper-level residents in the 2020-2021 academic year. This workshop included a didactic portion including a review of definitions, highlighting evidence for the health impact of ACEs on subsequent health outcomes, potential biological mechanisms and suggested solutions. This was followed by facilitated small group case-based discussions and generation of practical ideas for a more trauma-informed medical practice.

## Conclusions

In summary, trauma is a substantial public health issue and a driver of high-cost medical complexity and poor health outcomes. Internists are well-positioned to help lead efforts to deliver more compassionate and effective care if TIC training is introduced during and becomes natural in clinical practice. A single, brief TIC workshop was feasible, well-received, and associated with measurable improvements in IM residents’ self-reported knowledge, attitudes and skills on this topic.

## Appendices

Appendix A:

Health Equities Pathway (HEP) Content

Core Sessions 17-18

· Health Equity from Community Perspective: Bill Burman, MD*

· Patient Narrative of experience of poverty/mental illness: Facilitated by Sarah Stella, MD

· Patient cases from Social Work Perspective (housing, food resources): Veronica LynnLee, MSW^ & Rachel Slaughter, LCSW^#^

Dedicated Sessions 17-18

· Health Literacy: Gretchen Orosz, MD

· Using Interpreters in Clinical setting: Ellen Sarcone, MD & Andrea Salzberg, MD

· Unconscious bias and microaggression: Shanta Zimmer, MD

· Chronic pain, addiction and opioids: Kaylin Klie, MD

· Harm Reduction and HRAC^†^: Lisa Raville, Executive Director Harm Reduction Action Center

· Panel discussion on opioids and health equity: Lisa Raville, Nick Sarcone JD^‡^, Kaylin Klie MD

· Patient advocacy as a Physician and Legislator: Senator Irene Aguilar, MD

· Health Policy climate in Colorado: Ian Pelto, Colorado Health Institute

· State Capitol tour         

Core Sessions 18-19

· Homelessness, Health Outcomes and Housing First: Matt Mollica, Director of Housing Intake and Placement, Colorado Coalition for the Homeless

· How to become an Advocate: Smitha Chadaga, MD (Oregon Health Sciences University)

Dedicated Sessions 18-19

· Refugee 101: International Rescue Committee Representative

· Refugee Healthcare: Jamal Moloo, MD

· Issues for Physicians caring for Undocumented Immigrants/Know Your Rights: Arash Jahanian JD, Staff Attorney ACLU§ Colorado

· State Capitol advocacy session- Residents met with at least one legislator and prepped one page fact sheet advocating for current bill in Colorado legislature: Facilitated by Jerry Johnson, Anschutz Government Relations Lobbyist

· Trauma Informed Care Training Jennifer Perlman, PsyD^¶^ Coordinator of Trauma Informed Care for Colorado Coalition for the Homeless

*Medical Doctor

^Master of Social Work

^#^Licensed Clinical Social Worker

^†^Harm Reduction Action Center

^‡^Juris Doctor

^§^American Civil Liberties Union

^¶^Doctor of Psychology

Appendix B:

HEP Pre- and Post- Surveys

Residents’ Knowledge and Attitudes Regarding Trauma and Trauma Informed Care

PRE-SURVEY

We are interested in learning about your experiences caring for patients who have experienced trauma, and your experience regarding trauma informed care. Your responses to the following questions are confidential and will be utilized to improve curriculum in University of Colorado Internal Medicine Residency Program. Thank you for your participation!

Demographic Information

1. Please indicate your gender:

Male

Female

Other

Prefer not to say

2. Please indicate your level of training:

PGY-2

PGY-3

Prefer not to say

3. Have you received training on trauma informed care in the past?

Yes (If so, please outline where/when/description of training)

No

4. Are you in the Health Equity Pathway?

Yes

No

Content Questions

Please read each of the following statements and indicate to what extent you agree or disagree.

1. Trauma is prevalent among patients I treat.

Strongly Disagree                                     Neutral                                    Strongly Agree

            1                           2                             3                      4                         5

2. I can identify different types of trauma (e.g., developmental, single event trauma, cultural and intergenerational).

Strongly Disagree                                     Neutral                                    Strongly Agree

            1                           2                             3                      4                         5

3.     Trauma is distinct from everyday stress.

Strongly Disagree                                    Neutral                                     Strongly Agree

1                            2                              3                        4                         5

4.     Trauma impacts an individual’s ability to seek medical care.

Strongly Disagree                                     Neutral                                    Strongly Agree

1                      2                                  3                      4                         5

5.     I can identify specific instances where an understanding of trauma-informed care is useful in patient care.  

Strongly Disagree                                     Neutral                                    Strongly Agree

1                             2                             3                      4                          5

6.     I am comfortable inquiring about physical, emotional, and sexual abuse in my patients.

Strongly Disagree                                     Neutral                                    Strongly Agree

1                              2                               3                      4                            5

7.     I am able to recognize signs and symptoms of trauma, even if my patient does not disclose them to me.

Strongly Disagree                                     Neutral                                    Strongly Agree

1                             2                             3                      4                         5

8.     I routinely encourage patients to disclose what traumatic experiences they feel comfortable sharing.

Strongly Disagree                                     Neutral                                    Strongly Agree

1                       2                                    3                      4                         5

9.     I routinely ask patients how they cope with emotional responses that may result from traumatic experiences.

Strongly Disagree                                     Neutral                                    Strongly Agree

         1                        2                                   3                      4                         5

10.  I know what resources are available for patients after discussing his/her history of trauma, including coping strategies.

Strongly Disagree                                     Neutral                                    Strongly Agree

            1                           2                             3                      4                         5

11.  Patients who experience trauma have challenging medical and social issues which I can learn from.

Strongly Disagree                                     Neutral                                    Strongly Agree

            1                           2                             3                      4                         5

12.  Patients are personally responsible for the trauma they experience (e.g., substance use).

Strongly Disagree                                     Neutral                                    Strongly Agree

            1                           2                             3                      4                         5

13.  Patients who experience trauma frequently over-utilize health care resources.

Strongly Disagree                                     Neutral                                    Strongly Agree

            1                           2                             3                      4                         5

14.  Adverse life circumstances are likely to be responsible for a patient’s trauma.

Strongly Disagree                                     Neutral                                    Strongly Agree

            1                           2                             3                      4                         5

15.  Caring for patients with trauma is difficult and leads to burnout and compassion fatigue.

Strongly Disagree                                     Neutral                                    Strongly Agree

            1                           2                             3                      4                         5

16.  Patients who have experienced trauma may have difficulties adhering to medical therapies as prescribed.

Strongly Disagree                                     Neutral                                    Strongly Agree

            1                           2                             3                      4                         5

17.  Patients who leave medical care against medical advice might be exhibiting the effects of trauma.

Strongly Disagree                                     Neutral                                    Strongly Agree

            1                           2                             3                      4                         5

18.   I have heard of the Adverse Childhood Experiences (ACE) Study.                        

Yes                  No

19.  I have administered and assessed an Adverse Childhood Experiences (ACE) questionnaire in the past.

Yes                  No

Residents’ Knowledge and Attitudes Regarding Trauma and Trauma Informed Care

POST-SURVEY (1 week)

Thank you for participating in our trauma-informed care workshop. Your responses to the following questions are confidential and will be utilized to improve curriculum in University of Colorado Internal Medicine Residency Program. You will notice all questions are the same except for the open-ended questions at the end of the survey. We appreciate your time and thoughts!

POST-SURVEY (3 months)

You participated in a trauma-informed care workshop as a member of the Health Equities Pathway within the University of Colorado Internal Medicine Residency Training Program approximately three months ago. Please complete the following survey to regarding your thoughts and perceptions of trauma-informed care reflecting back on your experience during the workshop.

Demographic Information

1. Please indicate your gender:

Male

Female

Other

Prefer not to say

2. Please indicate your level of training:

PGY-2

PGY-3

Prefer not to say

3. Have you received training on trauma informed care in the past?

Yes  (If so, please outline where/when/description of training)

No

4. Are you in the Health Equity Pathway?

Yes

No

Content Questions

Please read each of the following statements and indicate to what extent you agree or disagree.

1. Trauma is prevalent among patients I treat.

Strongly Disagree                                     Neutral                                    Strongly Agree

            1                           2                             3                      4                         5

2.  I can identify different types of trauma (e.g., developmental, single event trauma, cultural and intergenerational).

Strongly Disagree                                     Neutral                                    Strongly Agree

            1                           2                             3                      4                         5

3.  Trauma is distinct from everyday stress.

Strongly Disagree                                    Neutral                                     Strongly Agree

         1                        2                                   3                      4                         5

4.  Trauma impacts an individual’s ability to seek medical care.

Strongly Disagree                                     Neutral                                    Strongly Agree

         1                        2                                   3                      4                         5

5.  I can identify specific instances where an understanding of trauma-informed care is useful in patient care. 

Strongly Disagree                                     Neutral                                    Strongly Agree

         1                        2                                   3                      4                         5

6.  I am comfortable inquiring about physical, emotional, and sexual abuse in my patients.

Strongly Disagree                                     Neutral                                    Strongly Agree

         1                        2                                   3                      4                         5

7.  I am able to recognize signs and symptoms of trauma, even if my patient does not disclose them to me.

Strongly Disagree                                     Neutral                                    Strongly Agree

         1                        2                                   3                      4                         5

8.  I routinely encourage patients to disclose what traumatic experiences they feel comfortable sharing.

Strongly Disagree                                     Neutral                                    Strongly Agree

         1                        2                                   3                      4                         5

9.  I routinely ask patients how they cope with emotional responses that may result from traumatic experiences.

Strongly Disagree                                     Neutral                                    Strongly Agree

         1                        2                                   3                      4                         5

10.  I know what resources are available for patients after discussing his/her history of trauma, including coping strategies.

Strongly Disagree                                     Neutral                                    Strongly Agree

            1                           2                             3                      4                         5

11.  Patients who experience trauma have challenging medical and social issues which I can learn from.

Strongly Disagree                                     Neutral                                    Strongly Agree

            1                           2                             3                      4                         5

12.  Patients are personally responsible for the trauma they experience (e.g., substance use).

Strongly Disagree                                     Neutral                                    Strongly Agree

            1                           2                             3                      4                         5

13.  Patients who experience trauma frequently over-utilize health care resources.

Strongly Disagree                                     Neutral                                    Strongly Agree

            1                           2                             3                      4                         5

14.  Adverse life circumstances are likely to be responsible for a patient’s trauma.

Strongly Disagree                                     Neutral                                    Strongly Agree

            1                           2                             3                      4                         5

15.  Caring for patients with trauma is difficult and leads to burnout and compassion fatigue.

Strongly Disagree                                     Neutral                                    Strongly Agree

            1                           2                             3                      4                         5

16.  Patients who have experienced trauma may have difficulties adhering to medical therapies as prescribed.

Strongly Disagree                                     Neutral                                    Strongly Agree

            1                           2                             3                      4                         5

17.  Patients who leave medical care against medical advice might be exhibiting the effects of trauma.

Strongly Disagree                                     Neutral                                    Strongly Agree

            1                           2                             3                      4                         5

18.   I have heard of the Adverse Childhood Experiences (ACE) Study.                        

Yes                  No

19.  I have administered and assessed an Adverse Childhood Experiences (ACE) questionnaire in the past.

Yes                  No

20.  What was the most important thing you learned from the trauma informed care workshop?

21.  What further training would you like to receive on this topic?

Appendix C

Non-HEP Survey

Residents’ Knowledge and Attitudes Regarding Trauma and Trauma Informed Care

NON-Health Equities Pathway Survey

We are interested in learning about your experiences caring for patients who have experienced trauma, and your experience regarding trauma informed care. Your responses to the following questions are confidential and will be utilized to improve curriculum in University of Colorado Internal Medicine Residency Program. Thank you for your participation!

Demographic Information

1. Please indicate your gender:

Male

Female

Other

Prefer not to say

2. Please indicate your level of training:

PGY-2

PGY-3

Prefer not to say

3. Have you received training on trauma informed care in the past?

Yes  (If so, please outline where/when/description of training)

No

4. Are you in the Health Equity Pathway?

Yes

No

Content Questions

Please read each of the following statements and indicate to what extent you agree or disagree.

1.     Trauma is prevalent among patients I treat.

Strongly Disagree                                     Neutral                                    Strongly Agree

            1                           2                             3                      4                         5

2.     I can identify different types of trauma (e.g., developmental, single event trauma, cultural and intergenerational).

Strongly Disagree                                     Neutral                                    Strongly Agree

            1                           2                             3                      4                         5

3.     Trauma is distinct from everyday stress.

Strongly Disagree                                    Neutral                                     Strongly Agree

1                            2                              3                        4                         5

4.     Trauma impacts an individual’s ability to seek medical care.

Strongly Disagree                                     Neutral                                    Strongly Agree

1                      2                                  3                      4                         5

5.     I can identify specific instances where an understanding of trauma-informed care is useful in patient care. 

Strongly Disagree                                     Neutral                                    Strongly Agree

1                             2                             3                      4                          5

6.     I am comfortable inquiring about physical, emotional, and sexual abuse in my patients.

Strongly Disagree                                     Neutral                                    Strongly Agree

1                              2                               3                      4                            5

7.     I am able to recognize signs and symptoms of trauma, even if my patient does not disclose them to me.

Strongly Disagree                                     Neutral                                    Strongly Agree

1                             2                             3                      4                         5

8.     I routinely encourage patients to disclose what traumatic experiences they feel comfortable sharing.

Strongly Disagree                                     Neutral                                    Strongly Agree

1                       2                                    3                      4                         5

9.     I routinely ask patients how they cope with emotional responses that may result from traumatic experiences.

Strongly Disagree                                     Neutral                                    Strongly Agree

         1                        2                                   3                      4                         5

10.  I know what resources are available for patients after discussing his/her history of trauma, including coping strategies.

Strongly Disagree                                     Neutral                                    Strongly Agree

            1                           2                             3                      4                         5

11.  Patients who experience trauma have challenging medical and social issues which I can learn from.

Strongly Disagree                                     Neutral                                    Strongly Agree

            1                           2                             3                      4                         5

12.  Patients are personally responsible for the trauma they experience (e.g., substance use).

Strongly Disagree                                     Neutral                                    Strongly Agree

            1                           2                             3                      4                         5

13.  Patients who experience trauma frequently over-utilize health care resources.

Strongly Disagree                                     Neutral                                    Strongly Agree

            1                           2                             3                      4                         5

14.  Adverse life circumstances are likely to be responsible for a patient’s trauma.

Strongly Disagree                                     Neutral                                    Strongly Agree

            1                           2                             3                      4                         5

15.  Caring for patients with trauma is difficult and leads to burnout and compassion fatigue.

Strongly Disagree                                     Neutral                                    Strongly Agree

            1                           2                             3                      4                         5

16.  Patients who have experienced trauma may have difficulties adhering to medical therapies as prescribed.

Strongly Disagree                                     Neutral                                    Strongly Agree

            1                           2                             3                      4                         5

17.  Patients who leave medical care against medical advice might be exhibiting the effects of trauma.

Strongly Disagree                                     Neutral                                    Strongly Agree

            1                           2                             3                      4                         5

18.   I have heard of the Adverse Childhood Experiences (ACE) Study.                        

Yes                  No

19.  I have administered and assessed an Adverse Childhood Experiences (ACE) questionnaire in the past.

Yes                  No

20.  What further training would you like to receive on this topic?
